# The Role of Immunogenetics in COVID-19

**DOI:** 10.3390/ijms22052636

**Published:** 2021-03-05

**Authors:** Fanny Pojero, Giuseppina Candore, Calogero Caruso, Danilo Di Bona, David A. Groneberg, Mattia E. Ligotti, Giulia Accardi, Anna Aiello

**Affiliations:** 1 Laboratory of Immunopathology and Immunosenescence, Department of Biomedicine, Neuroscience and Advanced Diagnostic, University of Palermo, 90134 Palermo, Italy; fanny.pojero@gmail.com (F.P.); giuseppina.candore@unipa.it (G.C.); mattiaemanuela.ligotti@unipa.it (M.E.L.); giulia.accardi@unipa.it (G.A.); 2 Department of Emergency and Organ Transplantation, University of Bari Aldo Moro, 70124 Bari, Italy; danilo.dibona@uniba.it; 3 Institute of Occupational, Social and Environmental Medicine, Goethe University Frankfurt, Theodor-Stern-Kai 7, 60596 Frankfurt, Germany; arbsozmed@uni-frankfurt.de

**Keywords:** AB0, COVID-19, HLA, immunogenetics, KIR, SARS-CoV-2

## Abstract

Coronavirus disease 2019 (COVID-19) is induced by SARS-CoV-2 and may arise as a variety of clinical manifestations, ranging from an asymptomatic condition to a life-threatening disease associated with cytokine storm, multiorgan and respiratory failure. The molecular mechanism behind such variability is still under investigation. Several pieces of experimental evidence suggest that genetic variants influencing the onset, maintenance and resolution of the immune response may be fundamental in predicting the evolution of the disease. The identification of genetic variants behind immune system reactivity and function in COVID-19 may help in the elaboration of personalized therapeutic strategies. In the frenetic look for universally shared treatment plans, those genetic variants that are common to other diseases/models may also help in addressing future research in terms of drug repurposing. In this paper, we discuss the most recent updates about the role of immunogenetics in determining the susceptibility to and the history of SARS-CoV-2 infection. We propose a narrative review of available data, speculating about lessons that we have learnt from other viral infections and immunosenescence, and discussing what kind of aspects of research should be deepened in order to improve our knowledge of how host genetic variability impacts the outcome for COVID-19 patients.

## 1. Introduction

Since the emergence of the first documented cases in December 2019, it soon became evident that subjects infected by SARS-CoV-2 experience a disease promptly named coronavirus disease 2019 (COVID-19) with dramatic interindividual variability in its clinical manifestations. In fact, COVID-19 may arise as an asymptomatic state as well as a panel of respiratory conditions ranging from mild flu-like symptoms (fever, fatigue and dry cough) to a severe respiratory disease (including pneumonia and dyspnoea) that may require hospitalization and may be exacerbated by cytokine storm, acute respiratory distress syndrome (ARDS), multi-organ and respiratory failure, with potentially fatal consequences [1,2,3,4]. In contrast to the SARS-CoV (-1)-caused SARS pandemic (2002/2003), recorded as the first pandemic of the 21st century, the SARS-CoV-2-caused COVID-19 pandemic was not stopped by public health preventive measures in its first season 2019/20. Instead, it enforced rapid development of vaccines. This was never accomplished for SARS-1 [5,6].

Epidemiological data demonstrated that clinically relevant variables like sex, age and comorbidities, as well as viral genome variants were not enough in explaining why subjects with apparently similar clinical history and pre-existent status develop such a different group of symptoms [2,4]. Thus, other factors must contribute to the pathogenesis of the disease, determining which patients will develop an asymptomatic infection and how serious the disease will be in symptomatic subjects [4,7]. Defining these factors will be especially advantageous to detect patients who may benefit from an early hospitalization and therapeutic intervention [2,3]. The attention was immediately captivated by those host genetic variants ruling the expression and characteristics of viral receptors and host enzymes involved in viral entry [1,3,7,8,9,10,11,12,13]. However, this represents only one of the elements that must be considered in the complex interplay involving the virus, the host respiratory tract–lung tissues, and the host immune system [2,7,14].

Host immune responses and immune-related symptoms are extremely variable between patients who have effective control of SARS-CoV-2, i.e., asymptomatic, and patients who are unable to control the virus, i.e., affected by severe COVID-19. This suggests that host immune dysregulation contributes to pathogenesis in some cases. However, it is not known if the development of a severe form of the disease is ruled by ether immune hyperactivity or by a failure to resolve the inflammatory response due to the ongoing viral replication and immune dysregulation. The correlation among cytokine levels, nasopharyngeal viral load and declining viral load in moderate cases suggests that the immune response is associated with the viral burden [15,16]. 

However, the genetic basis of the immune response, the focus of immunogenetics, may account for a notable part of the (still) unexplained interindividual disease variability and may provide the key to stratifying patients on the basis of the risk of developing severe symptoms according to the presence/absence of genetic variants [17]. In line with this hypothesis, a number of studies are currently ongoing in order to explore the genetic characteristics of COVID-19 patients as regards those genes that are involved in the setting/maintenance/control/switching off of the immune response [14,18]. 

## 2. AB0 Groups

The AB0 molecules represent complex membrane antigens widely expressed both on the surface of red blood cells (RBC) and many other cells, extending the importance and the clinical significance of the AB0 system beyond transfusion medicine. Historically, the AB0 phenotype was one of the first markers involved in cancer susceptibility. Evidence has since then accumulated that AB0 blood antigens could play a key role in various human diseases, although the data are not clear [19,20]. On this basis, it is not surprising that several studies have studied the associations between COVID-19 and the AB0 system.

The suspicion that the AB0 locus should be involved with the increased risk of developing a severe form of the disease arose from the observation that: (i) the frequency of blood group 0 among COVID-19 patients was lower vs. control subjects, whereas the frequency of blood group A among COVID-19 patients was higher vs. control subjects; (ii) the risk of infection was lower in blood group 0 subjects vs. non-0 blood groups; on the contrary, blood group A was associated with a higher risk of infection vs. non-A blood groups (Table 1) [21,22,23,24,25,26,27,28]. 

However, controversies still exist about the association of blood groups with the severity of the disease, with some reports documenting that blood group A is associated with an increased risk of death vs. non-A blood groups and blood group AB is associated with an increased risk of intubation and death vs. blood group 0, while other reports failed in reproducing the same observations or even in demonstrating an association between any blood group and disease severity (expressed variably as hospitalization, intensive care unit—ICU—admission, intubation, required proning, extracorporeal membrane oxygenation—ECMO) (Table 1) [21,23,26,27,30,31]. Obviously, the adoption of universal criteria to define the severity of COVID-19 would be extremely beneficial in terms of proper stratification of patients and reproducibility of the results.

A genome wide association study (GWAS) performed by The Severe Covid-19 GWAS Group on Italian and Spanish subjects demonstrated that the association of severe COVID-19 expressed as respiratory failure with rs657152 (A or C single nucleotide polymorphism (SNP)) at locus 9q34.2 (coinciding with the AB0 locus) was significant at the genome wide level, even after correction for sex and age. Moreover, blood-group-specific analysis corrected for age and gender showed a higher risk of experiencing respiratory failure in blood group A than in other blood groups and a protective effect in blood group 0 in comparison with other blood groups, but no association was detected with disease severity expressed as the need for mechanical ventilation (Table 1) [28]. 

In a subsequent study, the authors reported that the rs657152 SNP is in almost complete linkage disequilibrium (LD; D’ = 0.996, r^2^ = 0.97) with rs8176719 (c.259-1_259insG), whose deletion is the main determinant of group 0, allele AB0*O.01.01. However, no differences in the genotype and allele frequencies were detected comparing age matched COVID-19 patients who required hospitalization with healthy controls and comparing severe with non-severe COVID-19 patients (with severity defined as the need of critical care support—high-flow oxygen, positive-pressure ventilation, vasoactive drugs) [31]. 

A further source of heterogeneity may be represented by the choice of the pool of the analyzed patients. In a cohort of Italian transplanted and waiting for organ transplantation patients, blood group A was more frequent in COVID-19+ (45.5%) than COVID-19- patients (39.0%) but no difference in blood group A distribution was observed comparing dead and alive patients (Table 1); however, the authors do not provide readers with further details about the cause of death—if it was determined by COVID-19 related complications or not [29]. 

About possible mechanistic considerations, it would be worth deepening the observation that serum levels of inflammatory marker soluble E-selectin are higher in 0/0 individuals, whereas a single nucleotide polymorphism in A1 allele is associated with low levels of this inflammatory marker. Furthermore, subjects homozygous for G allele in rs657152 SNP, corresponding to blood type 0 carriers, showed higher interleukin-6 (IL-6) circulating levels respect to non-0 carriers [19]. Moreover, ABH oligosaccharide structures have been identified on the N-linked oligo-saccharide chains of von Willebrand factor (VWF) located in the A1 domain, which contains the binding site for platelet glycoprotein Ib. The VWF levels are approximately 25% higher in individuals who have a blood group other than 0, and it might depend on endothelial A, B glycosyltransferase enzymes generating A and B antigens on the existing VWF “H” oligosaccharides. In turn, this addition to VWF might influence its blood level, and hence plasma levels of factor VIII and finally coagulation [19,20]. However, these literature data appear to conflict with the results of association studies, putting the accent on the fact that well-controlled inflammation is not “per se” a negative phenomenon, but rather the necessary response of the immune system to pathogenic viruses or bacteria [32]. Instead, the hyper inflammation response may be revealed to be dangerous (see below cytokine storm). This hypothesis is strengthened by the data of the high healing rates in the centenarians, known to have a good control of the inflammatory response [33].

Finally, the association could rely on the increase in natural antibodies influencing the history of SARS-CoV-2 infection [21,23,26,28,34,35]. 

## 3. HLA

The human leukocyte antigen (HLA) system codes for cell membrane proteins responsible for regulating the immune system. HLA genes are highly polymorphic; the different alleles are involved in fine-tuning the acquired immune responses. HLA classes have different functions. Class I (HLA-A, -B and -C) antigens present peptides from inside the cell on the cell surface. If the cell is infected by a virus, peptides of viral origin will be presented so that the cell can be lysed by CD8+ cytotoxic lymphocytes. Class II (HLA-DRA, HLA-DRB1, HLA-DRB3–5, HLA-DQA, HLA-DQB, HLA-DPA and HLA-DPB) molecules present peptides from outside the cell (which is an Antigen Presenting Cell) to CD4+ helper lymphocytes, which in turn stimulate B lymphocytes to produce antibodies against that specific antigen. Class III encodes the components of the complement system and the proinflammatory cytokine tumor necrosis factor (TNF-α). The high polymorphism of class I and II molecules affects the peptide binding groove, since it varies the amino acid sequences that can be housed within the groove. Different HLA alleles exhibit different peptide binding repertoires. Therefore, it is not surprising that different infectious diseases are associated with different HLA antigens, which are responsible for different humoral or cellular immune responses against different viral epitopes [17].

Furthermore, regulation of class II gene expression has been claimed to play a role in susceptibility/resistance to HLA-associated diseases [36]. In cancer, HLA class I gene expression was frequently down-regulated at both protein and mRNA levels and hypermethylation of the promoter regions of the HLA-A, -B and -C genes is a major mechanism of transcriptional inactivation [37]. This mechanism allowing evasion from the cytotoxic response has been shown to be present in cell line infection by the Epstein-Barr Virus (EBV) [38]. However, to the best of our knowledge no study has demonstrated this possibility for the immune responses to SARS-CoV-2 yet. The association between HLA alleles and COVID-19 infection and severity was assayed in a number of studies using laboratory, ecological, and in silico approaches. This kind of intense research was pushed by the mentioned fundamental role of the HLA in immune response setting and in susceptibility to infections [14,39,40,41,42], and also by the reports documenting that monocytes exhibit a decrease in HLA-DR expression inversely correlated with severity—the more HLA-DR decreases, the more the severity increases. Interpreting this piece of data in the light of other clinical and molecular characteristics of SARS-CoV-2 infected subjects, the authors concluded that HLA-DR levels may mirror the balance between the inflammatory and immunosuppressive status of COVID-19 patients [43,44]. 

### 3.1. Incidence and Susceptibility

Using an ecological approach, it was demonstrated that B*44 and C*01 alleles (whose prevalence is higher in northern Italy) showed a positive log-linear correlation with COVID-19 incidence rate measured in close proximity to the date of the national outbreak, keeping their positive association with COVID-19 incidence in multivariable regression analysis also adjusted for regions (Table 2). 

On the contrary, HLA-B*14, B*18, and B*49 alleles (more frequent in southern Italy) showed an inverse log-linear correlation with COVID-19 incidence rate, but these associations lost their significance in the multiple regression model [39]. The authors underlined the fact that the product of HLA-C*01 allele represents the ligand of Natural Killer (NK) inhibitor receptors Killer Cell Immunoglobulin-Like Receptor (KIR) 2DL2 and KIR2DL3 [49,50], thus suggesting a possible role for HLA-C*01 carrying in establishing the entity of the immune response [39] (see below). In a Chinese study, HLA-B*15:27 and HLA-C*07:29 were more frequent in COVID-19 patients vs. control subjects, with the results staying significant after correction. However, the small sample size may hamper the proper interpretation of the results, as the authors warn (Table 2) [45]. An in silico study reported that among the compared alleles HLA-B*46:01 had the smallest predicted binding capacity for SARS-CoV-2 peptides and extremely low binding affinity measured for human coronavirus conserved peptides (defined as dissociation constant <500 nM), thus suggesting a possible role for HLA-B*46:01 in determining disease susceptibility [51,52], but this association was not confirmed by a following study on Hong Kong Chinese patients (Table 2) [46]. Furthermore, serotype HLA-B22 was found more frequently in COVID-19 Hong Kong Chinese patients vs. non-age matched controls but the association was not significant when the comparison was made against age matched controls [46]. Finally, it was demonstrated that countries where HLA-A*02:01 is the most frequent in the population had lower numbers of COVID-19 cases out of 10^6^ population vs. countries where HLA-A*24:02 or HLA-A*11:01 are the most frequent alleles, as confirmed at two different time points (April 2020 *p* = 0.009 and August 2020 *p* = 0.013) [53]. 

### 3.2. Disease Severity

As regards the association of HLA alleles with disease severity, published papers report conflicting results, mainly due to the lack of shared criteria for disease severity classification, and the differences in the choice of compared groups of subjects. No significant correlation was found between HLA-B27 status and COVID-19 severity evaluated on an arbitrary scale ranging from mild to life-threatening disease by a team of researchers studying northern American patients (Table 2) [48]. These results were independently confirmed by a report on Hong Kong Chinese patients finding no significant association between HLA-B27 serotype and lymphopenia or disease severity expressed as mild (mild symptoms up to mild pneumonia) or severe (dyspnoea, hypoxia, or >50% lung involvement on imaging)/critical (respiratory failure, shock, or multi-organ system dysfunction) (Table 2) [46]. Instead, another study performed on a cohort of COVID-19 patients with severe (respiratory impairment, requiring non-invasive ventilation) and extremely severe (respiratory failure, requiring invasive ventilation and ICU admission) disease showed that the frequency of HLA-B*27:07, -DRB1*15:01, -DQB1*06:02 was higher in the studied COVID-19 patients vs. a reference group representing the local Italian population (Table 2) [41]. On the contrary, the assessment of allelic distribution at the *HLA* loci (Classes I and II) in a group of Italian and Spanish patients showed no association with the need for mechanical ventilation (Table 2) [28]. Comparing a group of mild (non-experimenting pneumonia) and severe (including severe and critically ill subjects according to the Chinese Center for Disease Control and Prevention -CDC- criteria) Chinese patients, HLA-C*14:02, -B*51:01, and -A*11:01 were associated with the severity of the disease in logistic regression analysis also adjusted for age and gender, with the three alleles in strong linkage disequilibrium, thus representing a haplotype. As regards Class II HLA alleles, -DRB1*14:04, -DRB1*01:01, -DQA1*01:01 represented severity risk alleles, whereas -DPB1*03:01 and -DRB1*12:01 were protective alleles (Table 2) [10]. A possible correlation between in silico predictions and observed symptoms was provided by Iturrieta-Zuazo et al. The authors demonstrated that mild or asymptomatic patients (mild group) exhibited a significant greater number of viral peptides, making a comparison with both hospitalized patients (moderate group) and subjects requiring ICU admission and supportive care (severe group) by HLA locus (HLA-A, -B, -C) and by HLA Class I genotype (for HLA Class I, *p* < 0.0001 estimated on the number of tightly (dissociation constant <50 nM) and loosely (dissociation constant < 500 nM) binding peptides, in both severe vs. mild and moderate vs. mild) [54].

### 3.3. Mortality

Regarding the association between HLA alleles and death rates, data are extremely varied. A Spanish study failed in detecting differences in HLA allele distribution comparing COVID-19 patients and healthy controls. However, the authors noticed that the Acute Physiology and Chronic Health Evaluation (APACHE)-II score, Sepsis-related Organ Failure Assessment (SOFA) score, and frequency of HLA-A*11, HLA-C*01 and HLA-DQB1*04 were higher in non-surviving vs. surviving patients. In their binomial logistic regression models, all the three alleles preserved their association with mortality after controlling for SOFA, and HLA-A*11 and HLA-C*01 kept their association after controlling for APACHE-II (Table 2) [40]. In Mexico, a statistically significant correlation was documented between HLA-DRB1*01:01 allele and the predicted fatality rate in hospitalized patients (R = −0.44, *p* = 0.02) [55]. Similarly, analyzing the HLA haplotype distribution in three Italian zones (northern, central and southern regions), the haplotype HLA-A*01:01g-B*08:01g-C*07:01g-DRB1*03:01g showed a positive correlation and the haplotype HLA-A*02:01g-B*18:01g-C*07:01g-DRB1*11:04g and a negative correlation with both COVID-19 incidence (number of cases/100,000 inhabitants) and mortality (number of deaths/100,000 inhabitants) at three out of four the analyzed timepoints (the first time point was reported to have no significant results because it was too premature) [56]. HLA-A*01:01, -B*08:01, -C*07:01,-DRB1*03:01, -DQA1*05:01 and -DQB1*02:01 is a common Caucasoid haplotype called 8.1 ancestral haplotype (AH). Intriguingly, numerous genetic studies reported that individuals with 8.1 AH have a higher risk of specific autoimmune disorders than those without these alleles [57]. It is worth nothing that bivariate correlation analysis among the regional frequencies of HLA-A*01:01g-B*08:01g-C*07:01g-DRB1*03:01g haplotype and incidence and mortality showed a cluster distribution, with the northern regions having the highest haplotype frequencies and the highest incidence and mortality, the central regions recording intermediate and the southern regions exhibiting the lowest values. The opposite frame was reported for the haplotype HLA-A*02:01g-B*18:01g-C*07:01g-DRB1*11:04g [56]. However, the alleles analyzed in this study HLA-A*02:01, HLA-B*08:01, HLA-A*01:01, HLA-B*18:01, HLA-C*07:01 are predicted to exhibit a good viral antigen presenting capacity, with HLA-A*02:01 offering the highest numbers of both tightly (dissociation constant < 50 nM, peptide number 267) and loosely (dissociation constant < 500 nM, peptide number 795) SARS-CoV-2 peptides [51,56], thus making the relationship between COVID-19 death rates and in silico estimated HLA antigen presenting capacity less clear. Curiously, in countries where HLA-A*02:01 is the most frequent in the population, a significant correlation exists with mortality (expressed as deaths out of 10^6^ population) vs. countries where HLA-A*24:02 or HLA-A*11:01 are the most frequent alleles, as confirmed at two different time points (April 2020 *p* = 0.003 and August 2020 *p* < 0.001, respectively), excluding from further analysis that the number of recorded deaths accounts for the simple increase in COVID-19 cases regardless of the HLA genotype. The authors also predicted the functional performance of the three analyzed alleles, with HLA-A*02:01 having the lowest viral antigen-presenting capacity in comparison with HLA-A*24:02 and HLA-A*11:01, completely contradicting a previous paper reporting that the number of loosely+tightly binding peptides was higher for HLA-A*02:01 vs. both HLA-A*11:01 and HLA-A*24:02 [51,53,54]. 

### 3.4. Transplanted Patients

Transplanted patients, for whom HLA typing is speedily available, may represent an important source of information. In a cohort of Italian transplanted or waiting for organ transplantation patients, HLA-DRB1*08 was associated with a higher risk of infection and with a higher risk of death, with both observations keeping their significance in adjusted logistic regression analysis. Importantly, in an in silico prediction, none of the DRB1*08 alleles were able to bind with high affinity any of the viral peptides included in the simulation, raising questions about the functional performance of HLA-DRB1*08 in the case of SARS-CoV-2 infection (Table 1) [29]. Similarly, research performed in the UK showed that in a cohort of COVID-19+ transplanted (kidney or hematopoietic stem cells) and on the waiting list for solid organ transplantation patients, HLA-DQB1*06 was significantly associated with the risk of infection vs. a group of individuals on the renal transplant waiting list representing the local population (52.5% vs. 36%, respectively), as summarized in Table 2 [47].

### 3.5. Functional and Mechanistic Considerations

One report explored the association of HLA types with the immunophenotypic and functional characteristics of immune cells. HLA-A*02:01-restricted SARS-CoV-2-reactive CD8+ T cells can be detected at higher levels in COVID-19 patients vs. uninfected subjects, but their frequency is lower than that recorded for influenza or EBV-specific memory CD8+ T cells in SARS-CoV-2 uninfected subjects. In addition, in COVID-19 convalescent subjects these HLA-A*02:01-restricted SARS-CoV-2-reactive CD8+ T cells expressed granzymes and/or perforin, but were negative for CD38, HLA-DR, PD-1, and CD71 activation markers, while all these markers where positive in acute phase patients [58]. 

The lack of uniformity among the recalled reports could be easily explained. Indeed, several methodological problems can elucidate the discrepancies observed in studies concerning the association of HLA with diseases as follows: (I) insufficient sample sizes to detect differences in HLA antigen frequencies; (II) inadequate inclusion with inappropriate mixing of data (cohort effect) and inappropriate control matching, i.e., lack of proper selection from the same target population; (III) the different genetic backgrounds of the studied population; (IV) the lack of Bonferroni’s correction for multiple comparisons [59]. Differences in susceptibility/resistance to the same disease in populations can then be linked to the presence of different HLA subtypes in the various populations [60]. Moreover, caution should be adopted in the attempt to justify the observed correlation between HLA allele geographical distribution and COVID-19 incidence, prevalence and related mortality. In fact, in silico analysis of predicted HLA allele binding affinity must be validated in vitro together with the evaluation of the effect of the calculated binding affinity for each HLA allele on the increase in sustained immune responses and on cytokine synthesis control.

Concerning the meaning of the observed associations of HLA with COVID-19, it has to be pointed out that, in the last few years, it is becoming clear that Class I antigens can play a role as ligand for KIRs. Therefore, several observed associations of HLA class I antigen with COVID-19 might be explained by their role as ligands for KIR [61] (see above).

Finally, recent studies have investigated the role of nonclassical HLA class I HLA-E in the control of viral diseases. With only two alleles described, HLA-E shows a very low level of allelic variation. HLA-E is thus considered to play a role in both innate and adaptive immunity, by interacting with NK cells as well as presenting peptides to antigen-specific CD8^+^ T cells. The HLA-E alleles’ association with different viral infections seems to be discrepant. Each of the alleles can be advantageous for an individual or the entire population against a particular virus. Therefore, it has been assumed that the presence of both alleles in the gene pool can be beneficial for the survival of the population [62,63]. Thus, HLA-E should be an interesting new player in the field of immunology. However, to best of our knowledge no study has been performed on COVID-19 patients.

## 4. Other Immune Response Genes

COVID-19 severity and mortality may depend on attenuation or suppression of cytokines’ elicited pathways. In fact, SARS-CoV-2 blocks the proper synthesis of interferon-α (IFN-α) and interferon-β (IFN-β), thus impairing type I interferon signaling and leading to a suboptimal immune response [64,65]. The suppression of IFN-β synthesis is a mechanism shared with SARS-CoV, which exhibits a number of other ways to attenuate response to type I interferons, including interference with interferon receptor turnover or with the expression of interferon induced genes. However, these mechanisms have not been investigated deeply in SARS-CoV-2 infection yet [65]. Anyway, uncontrolled cytokine release and signaling may also cause life-threatening consequences.

Cytokine storm is an umbrella term encompassing a wide range of clinical and laboratory abnormalities. However, all cases involve elevated circulating cytokine levels, acute systemic inflammatory symptoms, and secondary organ dysfunction. A critical question concerns the factors that contribute to the severe cytokine storm-like phenotype observed in a small fraction of COVID-19 patients. Coexisting conditions such as hypertension, diabetes, and obesity are associated with more severe cases of COVID-19, possibly because of the pre-existing chronic inflammatory state or a lower threshold for the development of organ dysfunction from the immune response. Another prominent feature of COVID-19 severity is the association with advanced age [1,2,66,67]. Below the role of comorbidities, which tend to become more frequent during the aging process, a potential contributor to COVID-19 severity in older people may be represented by the subtle chronic inflammatory status called inflammaging (one of the three hallmarks of immunosenescence—an increase in serum cytokines impairing the effectivity of immune responses) and by the age-related progressive reduction in the ability to trigger effective antibody and cellular responses against infections and vaccinations [66,68,69,70,71]. Thus, immunosenescence represents a further potential contributor to the insurgence of fatal complications in COVID-19 [8,62,63]. Lymphopenia and accumulation of immune cells showing immunophenotypic and functional signs of exhaustion were commonly documented in COVID-19 patients, correlating with age and severity [43,68,72,73,74,75,76,77,78,79,80,81,82,83,84]. This strong resemblance to immunosenescence makes COVID-19 a possible scenario to deepen the knowledge about the molecular basis affecting normal immune cell metabolism/lifespan/turnover and to determine the importance of genetic variants and epigenetic modifications behind the appearance of inflammaging and immunosenescence [66,85,86,87,88]. Furthermore, the improvement of our understanding of the relationship between immunogenetics and immunosenescence (including inflammaging) would be crucial in the design of a therapeutic strategy specifically for older COVID-19 patients [66,68,69,89].

Several data suggest that cytokine storm may contribute to the pathogenesis of COVID-19. Serum cytokine levels that are elevated in patients with COVID-19-associated cytokine storm include IL-1β, IL-6, interferon γ-induced protein-10 (IP-10), TNF-α, interferon-γ (IFN-γ), macrophage inflammatory protein (MIP) -1α and -1β, and vascular endothelial growth factor (VEGF) [3,7,52,90,91,92,93]. 

Available data suggest that cytokine receptor antagonists might have a positive impact on survival of COVID-19 patients. IL-1 receptor antagonist Anakinra showed to be beneficial in terms of the need for mechanical ventilation and risk of death, despite larger studies being necessary to confirm if this agent is safe to be used alone or requires combination with other drugs such as glucocorticoids, and to estimate the exact entity of adverse effects like bacteremia. On the other hand, despite the association of high IL-6 levels with shorter survival and the evidence that laboratory findings of hyperinflammation (example, elevated values of C-reactive protein—CRP) and tissue damage predict worsening outcomes in COVID-19, data for anti–IL-6 receptor antibody therapies lack a uniform confirmation and/or are still insufficient, and are thus reported as conflicting in terms of mortality and hospitalization. This it is not surprising, since IL-1, IL-6 and other cytokines are potentially critical for both a healthy response to SARS-CoV-2 and a detrimental cytokine storm. Thus, completely blocking cytokine signaling might actually impair clearance of SARS-CoV-2, increase the risk of secondary infections, and lead to worse outcomes, as seen with influenza virus [16,94,95,96,97,98,99,100]. However, studies deeply dissecting the genetic basis of the insurgence of this phenomenon or the possible role of cytokine receptors in COVID-19 severity are still missing. 

### 4.1. Type I Interferons and Players of Their Molecular Pathways

Synthesis and release of type I interferons together with their signaling by binding to IFN-α receptor 1 (IFNAR1) and IFNAR2 are all fundamental steps in the defense process raised by viral RNA or DNA. These molecular pathways involve a number of actors, including toll like receptors (TLR), IFN regulatory factors (IRF), signal transducers and activator of transcription (STAT) 1 and 2, TIR-domain containing adaptor inducing IFN-β (TICAM1/TRIF), TANK binding kinase 1 (TBK10), TNF Receptor Associated Factor 3 (TRAF3) and Unc-93 homolog B1 (UNC93B1) [101,102,103,104,105,106,107,108,109,110,111,112]. 

A notable effort was made to analyze 12 autosomal loci (*STAT1* on chromosome 2, *TLR 3* on chromosome 4, *IRF7* and *UNC93B1* on chromosome 11, *TBK1* and *STAT2* on chromosome 12, *IRF9* and *TRAF3* on chromosome 14, *TICAM1/TRIF* and *IRF3* on chromosome 19, *IFNAR1* and *IFNAR2* on chromosome 21) and one X linked locus (NF-κB essential modulator—*NEMO/IKBKG*) in COVID-19 patients with life-threatening pneumonia vs. mild and asymptomatic COVID-19 cases (control group) [113,114]. The studied loci were connected to viral encephalitis, complications after measles, mumps, and rubella (MMR) vaccination and ARDS/critical influenza pneumonia [113,115,116]. As regards the 12 autosomal loci, a significant enrichment in what are predicted to be loss-of-function (pLOF) variants in patients vs. controls was detected under an autosomal-dominant (AD) mode of inheritance (Table 3). 

Using overexpression systems, 24 variants (including the pLOF ones) in *TLR3*, *UNC93B1*, *IRF7*, *IRF3*, *TICAM1/TRIF*, *TBK1*, *IFNAR1*, *IFNAR2* were demonstrated to be deleterious, since they were loss-of-expression, LOF or severely hypomorphic; of these 24 variants, four were autosomal-recessive (AR) deficiencies (homozygosity or compound heterozygosity for *IRF7*; homozygosity for *IFNAR1*) and 19 AD deficiencies (*TLR3*, *TICAM1*, *TBK1*, *IRF3*, *UNC93B1*, *IRF7*, *IFNAR1*, and *IFNAR2*). AR and AD IRF7-deficiency was associated with reduced levels of IRF7 expression on phytohemagglutinin (PHA) stimulated T cells. Plasmacytoid dendritic cells isolated from AR IRF7-deficient patients did not produce detectable type I or III IFNs on SARS-CoV-2 infection. Similarly, PHA stimulated T cells from a patient with AR IFNAR1 deficiency had impaired *IFNAR1* expression and response to type I IFNs. Then, the authors checked the functional consequences of *IRF7* and *IFNAR1* defects on SARS-CoV-2 susceptibility in vitro, taking into account that angiotensin-converting enzyme 2 (ACE2) works as a receptor for SARS-CoV-2, and that transmembrane protease serine protease 2 (TMPRSS2) processes SARS-CoV-2 S protein and activates viral entry [2,89]. Thus, they noticed that TLR3−/−, TLR3+/−, IRF7−/−, and IFNAR1−/− fibroblasts previously transduced with ACE2 and TMPRSS2 were more susceptible to SARS-CoV-2 infection in vitro vs. cells from control subjects but were rescued by transduction of wild type *IRF7* or *IFNAR1*. All these data suggest that genetic defects in the pathway of type I IFNs may be key determinant of COVID-19 severity [113]. 

Comforting these results, a recent study detected, validated and replicated the association between COVID-19 severity (i.e., admission to critical care) and rs2236757 (chr21q22.1) in the interferon receptor gene *IFNAR2* and rs10735079 in the gene cluster coding for interferon-inducible 2’-5’-Oligoadenylate Synthetase (*OAS*) 1, 2, and 3 (chr12q24.13) comparing severe COVID-19 patients with controls from the population [117] (Table 3). *OAS* genes encodes for enzymes that synthetize 2′,5′-oligoadenylate, leading to RNase L activation and dsRNA degradation [124]. Exon 3 and 3′ untranslated region (UTR) variants in OAS1 are reported to confer increased susceptibility to and protection against SARS, respectively [115]. 

Interferon-induced transmembrane protein 3 (IFITM3) is an endosomal antiviral protein which is upregulated as a consequence of type I and type II interferon signaling as well as by cytokines like IL-6. The rs12252(C) allele in *IFITM3* (chr. 11p15.5) was associated with influenza severity and mortality, and faster progression of HIV infection towards AIDS [125,126,127,128]. As regards COVID-19, the rs12252 (C) allele is considered a risk variant [120], especially as regards the homozygosity for the C allele, i.e., the (CC) genotype, in symptomatic cases [118,129]. As discussed in the previous sections, the adoption of uniform criteria to describe disease severity and the careful choice of the compared groups/populations are essential in order to obtain reproducible results. Comparing COVID-19 mild (fever, respiratory symptoms, and pneumonia at imaging) patients with severe (respiratory distress, blood oxygen saturation <93%, ratio of arterial oxygen pressure to fraction of inspired oxygen <300 mm Hg, respiratory failure with mechanical ventilation, shock, or other organ failure requiring intensive care in the intensive care unit) cases, an association between homozygosity for the C allele (CC vs. CT/TT) and disease severity was detected in logistic regression analysis adjusted for age (Table 3) [118]. Another study failed in demonstrating a significant association between carrying the C allele and disease severity intended as critical care support (including high-flow oxygen, positive-pressure ventilation or vasoactive drugs), but showed that carrying the C allele was associated with an increased risk of hospitalization comparing COVID-19 patients vs. controls—recruited before the pandemics—whose status about COVID-19 was unknown, with results keeping their significance in multiple logistic regression adjusted for age and sex (Table 3). No specific statistical analysis for the homozygosity for the C allele was carried [119]. 

Protein Activator of the Interferon-Induced Protein Kinase (PRKRA) together with IFN-induced, double-stranded RNA-activated protein kinase (PKR) is a long-time described mediator of interferon antiviral activity [130,131,132]. By whole exome sequencing and unbiased collapsing gene analysis, an Italian study identified *PRKRA* as one of the two protective genes (see below) comparing hospitalized COVID-19 patients with controls of unknown status. In this context, the deleterious variant was more frequent in COVID-19 patients than in controls [120]. 

### 4.2. Other Cytokines, Chemokines and Their Signaling Pathways

Data about promoter polymorphism -308 (G/A) (rs1800629) of *TNF-α* depict its involvement in the immune response set up and maintenance in different clinical scenarios, but the explanation of the related molecular mechanism is still pending. The minor allele A was described as associated with the risk of inflammatory disorders, sepsis, chronic obstructive pulmonary disease and asthma, whereas it confers protection against Dengue fever and has no effect on Kawasaki disease [133,134,135,136,137,138]. In addition, it exhibits a relation with increased serum TNF-α levels in asthma and not uniformly in cancer patients, but an analogous influence on transcription was not detected in other contexts like Alzheimer’s disease and generalized vitiligo [139,140,141,142,143]. A hospital-based case-control study comparing COVID-19 patients with controls described the frequency of −308 (G/A) *TNF-α* polymorphisms. The AA genotype and the A allele were more frequent in COVID-19 subjects vs. the control group (*p* = 0.019 and *p* = 0.005, respectively). Moreover, the AA genotype was associated with age > 60 years, increased degree lymphopenia, high CRP and serum ferritin (Table 3). The AA genotype was more frequent in severe (with any of the following: tachypnoea with a respiratory rate more than 30 cycle/min; PaO_2_ less than 300 mmHg; oxygen saturation below 93 at rest; shock; respiratory failure or other organ dysfunction) vs. mild cases (Table 3), with no GG genotype detected among severe patients. All dead subjects in this study carried the AA genotype [121]. 

Chemokine receptors are undoubtedly a keystone of immune cell trafficking; in particular, C-C Motif Chemokine Receptor 9 (CCR9) has a documented role in mediating immune cell localization in inflammatory disorders, C-X-C Motif Chemokine Receptor 6 (CXCR6) rules memory T cell recruiting in the airways, and X-C Motif Chemokine Receptor 1(XCR1), with its ligand X-C Motif Chemokine Ligand 1 (XCL1), has effects on the establishment of cytotoxic immune responses [144,145,146,147,148,149,150,151]. Thus, it would sound logical to suppose that any polymorphism impacting the transcription levels of these three receptors may affect the ability to mount an adequate immune response, also against airway pathogens. A GWAS revealed that the rs11385942 insertion–deletion GA or G variant at locus 3p21.31 was associated with COVID-19 related respiratory failure. The homozygosity for the risk allele GA was encountered in younger subjects vs. heterozygosity or homozygosity for the non-risk allele (59 years median age—interquartile range 49 to 68- vs. 66 years median age—interquartile range, 56 to 75; *p* = 0.005). The association locus included genes coding for chemokine receptors *CCR9*, *CXCR6*, and *XCR1* (plus the genes *SLC6A20*, *LZTFL1*, and *FYCO1*), and the risk allele GA of rs11385942 accounted for a reduction in the expression of CXCR6. Moreover, the frequency of the risk allele was associated with a higher risk of receiving mechanical ventilation, also after correction for sex and age (Table 3) [28]. More studies are necessary to deepen these results, also establishing a causal relationship between expression of chemokine receptors and history of the SARS-CoV-2 infection. Supporting this urgency, an Italian study focused on hospitalized COVID-19 patients looking for variants involved in viral infection, susceptibility or protection by whole exome sequencing; the authors detected variant rs1799864 of C-C Motif Chemokine Receptor 2 (CCR2) in 8 out of 35 patients and rs1800940 of CCR5 (both associated with protection against HIV) in 1 out of 35 patients [120,152,153]. Furthermore, in lung tissues, transcriptome-wide association demonstrated that predicted low levels of CXCR6 together with low expression of CCCR3 and high expression of CCR2 are associated with severe COVID-19 (defined as admission to critical care) vs. controls [117,154].

There is a general paucity of data regarding interleukin signaling pathways; however, interesting information may be extrapolated by more general studies. Products of the neighboring transmembrane protein 189 (TMEM189) and ubiquitin-conjugating enzyme E2 variant 1 (UBE2V1) genes are involved in IL-1 pathway, with variants in UBE2V1 also associated with HIV-1 acquisition risk [155,156,157,158]. Using a genome wide association approach on COVID-19 patients divided into a mild (asymptomatic+mild+moderate subjects) group and a severe (severe+critically ill subjects) group according to Chinese CDC criteria, a report documented that A allele frequency of intronic rs6020298 in *TMEM189–UBE2V1* (chr. 20q13.13) showed a significant association with both mild and severe conditions (Table 3) and a severity score estimated on the basis of age, gender and laboratory assessment (linear regression *p* = 1.1 × 10^–6^, *β* = 0.35). A similar association is shown by SNPs in linkage disequilibrium with rs6020298 (r^2^ > 0.8). The rs6020298 variant is an expression quantitative locus (eQTL) for TMEM189, with the A allele increasing *TMEM189* expression [10].

A number of results are already available to address future research in this direction, in the form of papers awaiting the peer review process. 

One preprint compares the frequency of rs1800796 and rs1800795 of IL-6, rs2228145 of IL-6R, rs1800896 and rs1800871 of IL-10, rs2275913 of IL-17A and rs76378 of IL-17F as recorded in previous studies with the prevalence/10^6^ population and mortality/10^6^ population of COVID-19 in China, Japan, India, Iran, Spain, Italy, Mexico, Netherlands, Sweden, Turkey, Finland, Brazil, Czechia, Russia, Poland, suggesting a possible role for rs2275913 of IL-17A in COVID-19 prevalence and mortality [159,160,161,162]. This piece of data is particular intriguing, assuming that the analyzed polymorphism is reported as associated with susceptibility to cutaneous and airway infections, ARDS and asthma [163,164,165,166,167,168,169].

Another preprint leading with supervariants involved in COVID-19 mortality focuses on the possible role of rs60811869, an eQTL of gene of the E3 ubiquitin ligase WD Repeat And SOCS Box Containing 1 (*WSB1*), in determining death rates among COVID-19 patients [170,171]. WSB1 is a particularly promising candidate, being involved in hormone homeostasis and hypoxia, as well as in the maturation of IL-21 receptor with IL-21 being a key player in humoral immunity and in the formation of the immune memory [172,173,174,175,176]. 

### 4.3. Other Genetic Determinants of Establishment/Maintenance/Resolution of the Immune Response and Antigen Presentation

Among dipeptidyl peptidase (DPP) family members, *DPP9* on chromosome 19 seems to be implicated in macrophage differentiation and apoptosis [177,178]. In addition, it is involved in idiopathic pulmonary fibrosis [179]. Variant rs2109069 in the *DPP9* gene is associated with disease severity in a cohort of UK patients (Table 3) [117]. Instead, *DPP7* (9q34.3) is essential for the maintenance of quiescence in lymphocytes [180,181,182] and its expression is associated with influenza vaccination response [183]. Using a pedigree approach, a study demonstrated that a 1-bp insertion in *DPP7* (rs11391519), disrupting gene transcription, was present in asymptomatic subjects [10]. 

Looking for monogenic effects determining COVID-19 severity, the same group reported the rs143359233 splice acceptor variant in golgin subfamily A 3 (*GOLGA3*, chr. 12q24.33) in critically ill patients (defined as subjects with one of the following conditions: respiratory failure and requesting mechanical ventilation, shock, failure of other organs and requesting intensive care monitoring) [10,184]. This is an interesting piece of data, since golgin family members are involved in nuclear transport, and another variant in *GOLGA3* (rs12282) is associated with response to smallpox vaccine [185,186]. Intriguingly, this does not seem to be the only member of the golgin family involved in defining the history of SARS-CoV-2 infection. In fact, through the single variant association test to compare COVID-19 hospitalized cases and controls, missense variant rs200975425 in golgin A8 family member B (*GOLGA8B* in15q14) emerged as associated with infection susceptibility [10,187,188,189]. 

Resident macrophages engaged in the fight against SARS-CoV-2 may be influenced by genetic variants ruling polarization—thus tissue macrophages’ functions. Macrophage stimulating 1 receptor (*MST1R* in 3p21.31) is expressed on macrophages and involved in their polarization [190,191,192,193,194,195]. In unrelated samples Wang et al. identified a loss of function variants in *MST1R* (hg38 chr3:49896789_C/T) in asymptomatic and mild subjects comparing them with moderate, severe and critically ill patients (all categories defined according to the Chinese CDC criteria) [10]. 

Antigen presentation by HLA-DR may be a further potential pattern whose molecular defects may lead to an incomplete or insufficient response in COVID-19 patients. Expression of lysosomal localized Lysosomal Protein Transmembrane 4 β (*LAPTM4B* in 8q22.1), a lysosomal regulator of autophagy and an essential partner for endosomal transporters is finely regulated, since its overexpression causes enlargement of lysosomes [26,196,197,198,199]. LAPTM4B is the second gene identified by an Italian study for which deleterious variants were reported at higher frequencies in COVID-19 patients than in controls (Table 3) [120]. 

Other candidate genes may be involved in immune cell activation at various levels, as the pro-inflammatory allele ε4 (apoliprotein E in 19q13.32) *ApoE* gene is known to be involved in the pathophysiology of age-related inflammatory diseases as well as in susceptibility to infectious diseases [200,201]. In a logistic regression model adjusted also for age and sex, the *ApoE* ε4ε4 homozygous genotype was found to be associated with an increased risk of developing COVID-19 vs. ε3ε3 homozygotes (Table 3), preserving its statistical significance after the exclusion of participants related to the third degree or closer, and patients carrying ε4 with dementia, hypertension, cardiovascular disease, and type 2 diabetes. In this study, disease positivity for COVID-19 was interpreted as a synonym of severity, given that testing was restricted to hospitalized symptomatic patients [14,122]. The same authors later expanded their analysis and noticed that carrying ε4 represents a susceptibility factor (Table 3), but only the results for ε4ε4 genotype kept their statistical significance after the exclusion of patients carrying ε4 with dementia, hypertension, cardiovascular disease, and type 2 diabetes. Similarly, comparing COVID-19 patients who died with subjects from the UK biobank excluding COVID-19+ surviving subjects, the *ApoE* ε4ε4 homozygous genotype was found to be significantly associated with an increased risk of death vs. ε3ε3 homozygotes (Table 3) even after the exclusion of patients carrying e4 with dementia, hypertension, cardiovascular disease, and type 2 diabetes [123].

**Figure 1:**
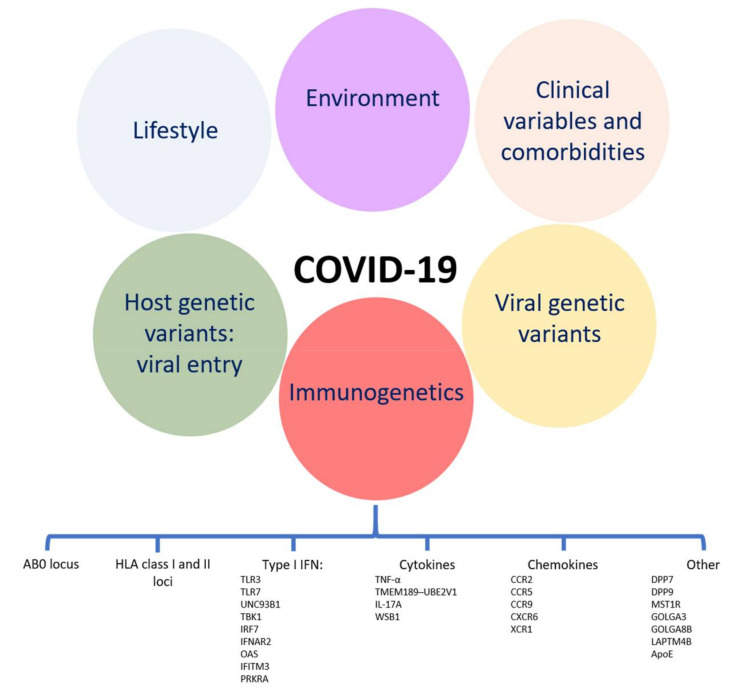
Scheme of factors influencing susceptibility to and severity of COVID-19. “Lifestyle” refers to habits (in terms of quality of nutrition, physical activity, drug consumption, smoking) and to socio-economic parameters (access to the health care system, level of education, access to high quality medical information), i.e., the social determinants of health. “Environment” (intended as exposure to contaminants, pollution, and non-ionizing radio frequency, but also as exposure to multiple pathogens) is relevant since it mechanistically modifies the immune response [202,203,204,205,206,207,208,209,210]. Viral genetic variants and their effects on virulence/COVID-19 severity are also a matter of intense research [211,212,213].

Figure 1 summarizes immunogenetic factors discussed in the present review [202,203,204,205,206,207,208,209,210,211,212,213].

## 5. Discussion

The continuously updated epidemiological and molecular data tell a story of a pandemic still lacking universally shared treatment protocols [2,214,215]. It is pretty evident that a number of factors contribute to determine SARS-CoV-2 infection susceptibility and COVID-19 severity (Figure 1), and that such a high number of new infections/hospitalized cases put extreme pressure on the global web of health care systems, creating the premises for the urgent need for a shared scheme to face the pandemic [2,214,216]. Together with public health measures to control the spread of the infection, stratifying patients according to the risk of developing a life-threatening disease could be a winning strategy to ensure the highest reachable survival rates while waiting for the completion of a global vaccination plan or the introduction of SARS-CoV-2 specific antiviral drugs [2,89,215]. The elaboration of an algorithm to calculate the probability of requiring hospitalization and to predict the efficacy and safety of early treatment/targeted treatment is an attractive perspective. Current efforts in this sense are focused on a number of predictors among clinical and laboratory evidence [1,217]. As demonstrated by experimental data, in this process the impact of host genetics, and—even better—immunogenetics should be surely included, together with gender related immunogenetic differences. 

Although SARS-CoV-2 may infect people regardless of age, ethnic group or sex, male older subjects have been identified as a high-risk group regarding the clinical outcome of the disease, both for developing severe pneumonia with respiratory distress and death [1,2,67]. The reason behind the increased severity of the disease in the male older population is currently an unanswered question. A sex-specific GWAS performed in the UK showed no sex specific association [117]. However, it has been demonstrated that a more rapid aging of the immune system occurs in men than women. In fact, the strength and types of immune responses are different between men and women. Oestrogens promote while androgens suppress immune responses in the case of infections, vaccination and autoimmunity. On these grounds, men are more susceptible to many infections, while women suffer more from infectious diseases with enhanced immunopathological impact as well as from autoimmune diseases [71]. Peripheral blood mononuclear cells of men and women significantly differ after the age of 65, contradicting expectations related to declining sex hormones. Annotation of sex-biased loci reveal that older women have higher genomic activity for adaptive cells and older men have higher activity for monocytes and inflammation [218,219]. 

In the case of COVID-19, some authors proposed that polymorphism in *ACE2* gene (located on chromosome X) coding and regulatory regions may partially explain such a sex-connected variability [11]. However, other factors may be involved, possibly including biochemical patterns ruling natural defenses against viruses (for example the sex related differences in H_2_S synthesis) as well as genes involved on immune responses located on chromosome X like CD40 ligand (*CD40L*), *TLR7*, *TLR8*, forkhead box p3 (*FOXP3*) and C-X-C Motif Chemokine Receptor 3 (*CXCR3*) [219,220,221,222,223,224,225]. Supporting this hypothesis, a study performed on two unrelated families (each one with a pair of male brothers with severe COVID-19) identified two variants in *TLR7*: (I) a pLOF variant NM_016562.3 c.2129_2132del, p.(Gln710Argfs*18) in the first family and (II) a missense variant NM_016562.3 c.2383G>T, p.(Val795Phe) in the second family. These variants significantly impaired the increase in *TRL7* transcription, the upregulation of type I IFN-related genes interferon β 1 (*IFNB1)*, *IRF7* and ubiquitin-like modifier ISG15 (*ISG15*) in the TLR7 pathway, and the IFN-γ production after stimulation with imiquimod [226,227,228]. 

The sex effect may also be the result of an interplay between non-immune oestrogen/progesterone/androgen functions and other immune causes. Intriguingly, a significant association was described between blood type A and frequency of COVID-19 in the female subgroup (*p* = 0.02, OR = 1.56, 95% CI = 1.08–2.27) but not in the male subgroup of analyzed subjects (*p* = 0.51, OR = 1.14, 95%CI = 0.78–1.67) in a cohort of gender-stratified Chinese controls and COVID19+ patients [24]. 

In addition, the role of ethnic dependent differences in the frequency of variants in immunity ruling genes would be worth deeper investigation. In this sense, the choice of technical strategies to investigate the immunogenetic diversity is crucial in order to obtain the deepest pieces of information and to predict the course of the disease. GWAS is surely a helpful approach in the identification of those ethnic groups showing an increased susceptibility to SARS-CoV-2 infection/life threatening COVID-19. GWAS are frequently performed on nationally available pools of patients [28,117,171]. It would be highly recommendable that the impact of ethnicity related genetic diversity is always deeply investigated [107], even when the chosen patients appear to be homogeneous for their ethnic background [28,171]. All GWAS data should be confirmed on larger/cross national scales, stratifying the obtained results on the basis of ethnic differences of the studied populations, thus in terms of genetic background.

Future analysis should include genes that have not been studied yet, such as, for example, KIRs and immunoglobulin G heavy chain (GM). KIRs, expressed on the membrane of NK cells and a minority of T lymphocytes, regulate the killing function of these cells by interacting with specific amino acid motifs (public epitopes) carried by some HLA class I molecules and expressed on their targets. The KIR gene complex is characterized by multiple gene-content haplotypes, i.e., it is polygenic and some genes are polymorphic. KIRs are able to detect cells infected by viruses and transformed cells by binding the different class I allelic variants. Most inhibitory KIRs specifically recognize sets of HLA class I alleles. Specific combinations of KIRs with their cognate HLA ligands have been associated with onset and severity of infectious diseases [229,230,231]. This supports the role for HLA class I diversity in the innate immune response in addition to the documented acquired immune response. GM allotypes contribute to the inter-individual differences in the magnitude of immune responsiveness. GM allotypes have been shown to be associated with immune responsiveness to several major infectious pathogens and with survival of epidemics [231]. 

Furthermore, another type of immune response control also deserves in-depth studies in relation to COVID-19: the so-called trained immunity. Indeed, exposure to selected vaccines, such as bacille Calmette—Guérin (BCG) or microbial components, can increase the baseline tone of innate immunity and trigger pathogen-agnostic antimicrobial resistance. Such epigenetics training is directly relevant to resistance against infectious diseases, including COVID-19 and several trials to determine whether BCG can help prevent or ameliorate COVID-19 are under way [232].

## 6. Conclusions

Experimental data underline the contribution of immunogenetics in determining the susceptibility to SARS-CoV-2 infection as well as severity and mortality of COVID-19. Some of the involved genes and loci have already been analyzed in literature for their involvement in other infectious processes (as happened for example for AB0 locus, HLA genes, sequences regulating the expression of/coding for cytokines and chemokines); others are less investigated and may open the space for mechanistic studies (for example, Golgin and DPP genes). The most evident conclusion is that available reports are not always reproducible, and no definitive conclusions may be inferred since a lack of uniformity hampers the elaboration of a definitive cause–effect relationship. This situation may depend on differences in the experimental settings and adopted criteria to stratify the analyzed population, as well as on other factors influencing the genetic background of the immune response, such as environment and lifestyle (also accounting for epigenetics modulation).

A huge comprehension of the impact of genetic/epigenetic variants on immune mechanisms would offer the chance to: (i) identify those molecular pathways that are impaired in the setting of the immune response against SARS-CoV-2, leading to a more severe disease and to a more difficult resolution of the infection; (ii) concentrate the efforts on the design of a personalized approach for those patients presenting variants associated with a notable risk of altered lymphocyte distribution, uncontrolled or suboptimal immune response and cytokine storm syndrome; (iii) detect molecular defects common to other diseases that may be considered as possible targets in terms of drug repurposing [10,12,89,233,234,235,236,237]. This specific pandemic situation requires a global coordinated approach to face the disease in a short time with the most limited loss of human lives. To reach such an ambitious and necessary goal, a priority will be the setting of shared criteria to stratify patients according to their clinical presentation (i.e., COVID-19 severity) and the prompt validation of in silico predictions, both of these representing a powerful tool to guarantee reproducibility of results and proper interpretation of epidemiological and experimental data.

## Figures and Tables

**Table 1:** List of the associations between blood groups and variants at the AB0 locus with COVID-19 susceptibility and severity.

Group/Locus	Variant	Outcome	*p*	OR (95% CI)	RR (95%CI)	Refs.
A						
		Susceptibility	0.027	1.21 (1.02–1.43)		[21]
		Susceptibility	* 0.04	* 1.33 (1.02–1.73)		[24]
		Susceptibility	0.0024	1.23 (1.08–1.41)		[25]
		Susceptibility	<0.001		1.09 (1.02–1.13)	[27]
		Susceptibility ^(1)^	<0.001	1.249 (1.114–1.440)		[26]
		Susceptibility ^(2)^	0.03	1.3 (1.02–1.66)		[29]
		Respiratory failure	* 1.48×10^−4^	* 1.45 (1.20–1.75)		[28]
		Mortality	0.008	1.482 (1.113–1.972)		[21]
AB						
		Susceptibility	* 0.035	* 1.37 (1.02–1.83)		[30]
B						
		Susceptibility	* 0.004	* 1.28 (1.08–1.52)		[30]
0						
		Susceptibility	<0.001	0.67 (0.60–0.75)		[21]
		Susceptibility	0.0006	0.787 (0.69–0.90)		[25]
		Susceptibility	<0.001		0.87 (0.82–0.91)	[27]
		Susceptibility	* 0.007	0.84 (0.75–0.95)		[30]
		Susceptibility ^(1)^	<0.001	0.699 (0.635–0.770)		[26]
		Respiratory failure	* 1.06×10^−5^	* 0.65 (0.53–0.79)		[28]
		Mortality	0.014	0.660 (0.479–0.911)		[21]
Locus AB0						
	rs657152 (A)	Respiratory failure	* 5.35×10^−7^	* 1.39 (1.22–1.59)		[28]

Group/locus, AB0 blood group or locus; variant, name of the polymorphism at the indicated locus (allele); outcome, type of assayed outcome; *p*, *p* value (corrected *p* values are preceded by a *); OR (95% CI), odds ratio (95% confidence interval) for the analysed blood group or variant (adjusted OR are preceded by a *); RR (95% CI), relative risk (95% confidence interval) for the analysed blood group or variant; Ref, references. (1) meta-analysis; (2) in transplanted patients.

**Table 2:** Association between human leukocyte antigen (HLA) class I and II allele/serotype/haplotype with COVID-19 incidence, susceptibility and severity.

Variable	*Allele*/Serotype	*p*	OR (95%CI)	Regression Coefficient	Growth Rate (95%CI)	Refs.
Incidence	B*44	0.05		0.1484	1.16 (1–1.35%)	[39]
	C*01	0.042		0.1747	1.19 (1.01–1.41%)	[39]
						
Susceptibility	B*15:27	0.030	3.59 (1.72–7.50) ^§^			[45]
	B46	n.s.				[46]
	B22	0.032	1.71 (1.23–2.38) ^§^			[46]
	C*07:29	0.025	130.20 (5.28–3211) ^§^			[45]
	DQB1*06	0.0468 ^#^	1.96 (1.19–3.22) ^#§^			[47]
	DRB1*08	0.010 ^#^	1.814 (1.151–2.860) ^#^			[29]
						
Severity	*A*	n.s.				[28]
	A*11:01	0.008	2.33			[10]
	B22	n.s.				[46]
	B27	n.s.				[46,48]
	B*27:07	* 0.004				[41]
	B46	n.s.				[46]
	B*51:01	0.007	3.38			[10]
	*C*	n.s.				[28]
	C*14:02	0.003	4.75			[10]
	DPB1*03:01	0.037	0.09			[10]
	DQA1*01:01	0.039	6.05			[10]
	*DQB1*	n.s.				[28]
	DQB1*06:02	0.016				[41]
	*DRB1*	n.s.				[28]
	DRB1*01:01	0.02	13.7			[10]
	DRB1*12:01	0.045	0.18			[10]
	DRB1*14:04	0.01	15.1			[10]
	DRB1*15:01	0.048				[37]
						
	A*11	0.04 ^(1)^	7.693 (1.063–55.65) ^(1)^			[40]
		0.02 ^(2)^	11.858 (1.524–92.273) ^(2)^			[40]
Mortality	C*01	0.04 ^(1)^	11.182 (1.053–118.7) ^(1)^			[40]
		0.02 ^(2)^	17.604 (1.629–190.211) ^(2)^			[40]
	DQB1*04	0.03 ^(1)^	9.963 (1.235–80.358) ^(1)^			[40]
	DRB1*08	0.01^ #^	8.6 (1.7–43.9) ^#^			[29]

Variable, type of variable considered in the statistical analysis; allele/serotype, HLA alleles or serotypes; *p*, *p* value (corrected *p* values are preceded by a *); OR (95% CI), odds ratio (95% confidence interval) for the analyzed allele/serogroup/haplotype; regression coefficient, regression coefficient at regression analysis; growth rate (95% CI), growth rate (95% confidence interval) for the analyzed allele/serogroup; Ref, references; n.s. not significant. Alleles are reported in italics. The listed statistics, coefficients and *p* values are corrected. Unadjusted statistics are indicated by a ^§^. # in transplanted patients; (1) after controlling for Sepsis-related Organ Failure Assessment (SOFA)—see text for details. (2) after controlling for the Acute Physiology and Chronic Health Evaluation (APACHE)-II—see text for details.

**Table 3:** List of genetic variants influencing immune responses associated with COVID-19 susceptibility, severity and mortality.

Gene/Locus	Variant/ Position	Reference/ Other Allele	Altered/ Risk Allele	Protein Variant	Type	*p*	OR (95%CI)	Outcome	Refs.
*TLR3*	^§^ 187003852	AT	A	p.Ser339fs	pLOF	0.01	8.28 (1.04–65.64)	severity	[113]
*TLR3*	^§^ 187005146	G	A	p.Trp769 *	pLOF				
*UNC93B1*	^§^ 67770598	C	A	p.Glu96 *	pLOF				
*TBK1*	^§^ 64875731	C	T	p.Arg308 *	pLOF				
*IRF7*	^§^ 615095	A	C	p.Arg7fs	pLOF				
*IRF7*	^§^ 614300	G	A	p.Gln185 *	pLOF				
*IRF7*	^§^ 613966	CGGGCTGGGGCCCG	C	p.Pro246fs	pLOF				
*IRF7*	^§^ 613353	G	GC	p.Pro364fs	pLOF				
*IFNAR2*	^§^ 34621038	AGATTGTTGGTTTT	A	p.Glu140fs	pLOF				
*IFNAR2*	rs2236757	G	A			4.99 × 10^−8^	1.28	severity	[117]
*OAS3*	rs10735079	G	A			1.65 × 10^−8^	1.29	severity	[117]
*IFITM3*	rs12252	T	C			^#^ 0.0093	^#^ 6.37	severity	[118]
						0.025	1.93 (1.09–3.46)	severity	[119]
*PRKRA*				I226N		0.02		severity	[120]
*TNF-α*	rs1800629	G	A			° <0.001		age > 60	[121]
						° <0.001		lymphopenia	[121]
						° 0.009		high CRP	[121]
						° <0.001		high ferritin	[121]
						° <0.001		severity	[121]
						^†^ 0.045		severity	[121]
3p21.31	rs11385942	G	GA			1.15 × 10^−10^	1.77 (1.48–2.11)	respiratory failure	[28]
						0.003	1.56 (1.17–2.01)	mechanical ventilation	[28]
*TMEM189UBE2V1*	rs6020298	G	A			4.1 × 10^–6^	1.2	severity	[10]
*DPP9*	rs2109069	G	A			3.98 × 10^−12^	1.36	severity	[117]
*GOLGA8B*	rs200975425	C	T			9.4 × 10^–10^	5.4	susceptibility	[10]
*LAPTM4B*				P219L		0.029			[120]
				P220L					
				I109F					
				P50T					
*ApoE*		e3	e4			1.19 × 10^–6^	2.31 (1.65–3.24)	severity	[122]
						^ 0.009	^ 1.20 (1.05–1.37)	severity	[123]
						3.24 × 10^−9^	2.24 (1.72–2.93)	severity	[123]
						1.22 × 10^–6^	4.29 (2.38–7.72)	mortality	[123]

Gene/locus, analyzed gene or locus; variant/position, variant name, or position of the variant on reference human genome; reference/other allele., reference allele or other allele; altered/risk allele, altered allele or risk allele; protein variant, changes in the aminoacidic sequence; *p*, *p* value (all the reported *p* values are corrected); OR (95% CI), odds ratio (95% confidence interval); outcome, type of the assayed outcome; Ref, references. § GRCh37; # homozygosity for the risk allele; ° homozygosity for the risk allele vs. homozygosity for the other allele; † homozygosity for the risk allele vs. heterozygosity for the risk allele; ^ heterozygosity for the risk allele vs. homozygosity for the other allele.

## Abbreviations

ACE2	Angiotensin-converting enzyme 2
AD	Autosomal-dominant
AH	Ancestral haplotype
AIDS	Acquired immunodeficiency syndrome
APACHE	Acute Physiology and Chronic Health Evaluation
ApoE	Apoliprotein E
AR	Autosomal-recessive
ARDS	Acute respiratory distress syndrome
CD40L	CD40 ligand
CDC	Centers for Disease Control and Prevention
CCR2	C-C Motif Chemokine Receptor 2
CCR3	C-C Motif Chemokine Receptor 3
CCR5	C-C Motif Chemokine Receptor 5
CCR9	C-C Motif Chemokine Receptor 9
COVID-19	Coronavirus disease 19
CRP	C-reactive protein
CXCR3	C-X-C Motif Chemokine Receptor 3
CXCR6	C-X-C Motif Chemokine Receptor 6
DPP	Dipeptidyl peptidase
EBV	Epstein-Barr Virus
ECMO	Extracorporeal membrane oxygenation
eQTL	Expression quantitative locus
FOXP3	Forkhead box p3
GM	Immunoglobulin G heavy chain
GOLGA3	Golgin subfamily A 3
GOLGA8B	Golgin A8 family member B
GWAS	Genome wide association study
HIV	Human immunodeficiency virus
HLA	Human Leukocyte Antigens
ICU	Intensive care unit
IFITM3	Interferon-induced transmembrane protein 3
IFNB1	Interferon β 1
IFN-α	Interferon-α
IFN-β	Interferon-β
IFN-γ	Interferon-γ
IFNAR1	IFN-α receptor 1
IFNAR2	IFN-α receptor 2
IL-1β	Interleukin-1β
IL-6	Interleukin-6
IL-10	Interleukin-10
IL-17A	Interleukin-17A
IL-17F	Interleukin-17F
IL-21	Interleukin-21
IP-10	Interferon γ-induced protein-10
IRF	IFN regulatory factors
ISG15	Ubiquitin-like modifier ISG15
KIR	Killer Immunoglobulin Receptors
KIR2DL2	Killer Cell Immunoglobulin-Like Receptor 2DL2
KIR2DL3	Killer Cell Immunoglobulin-Like Receptor 2DL3
LAPTM4B	Lysosomal Protein Transmembrane 4 β
MIP-1α	Macrophage inflammatory protein 1α
MIP-1β	Macrophage inflammatory protein 1β
MMR	Measles, mumps, and rubella
MST1R	Macrophage stimulating 1 receptor
NEMO/IKBKG	NF-κB essential modulator
NK	Natural Killer
OAS	2’-5’-Oligoadenylate synthetase
PHA	Phytohemagglutinin
PKR	IFN-induced, double-stranded RNA-activated protein kinase
pLOF	Predicted to be loss-of-function
PRKRA	Protein Activator of The Interferon-Induced Protein Kinase
RBC	Red blood cells
SNP	Single nucleotide polymorphism
SOFA	Sepsis-related Organ Failure Assessment
STAT1	Signal transducer and activator of transcription 1
STAT2	Signal transducer and activator of transcription 2
TBK1	TANK binding kinase 1
TICAM1/TRIF	TIR-domain containing adaptor inducing IFN-β
TLR	Toll like receptor
TMEM189	Transmembrane protein 189
TMPRSS2	Transmembrane protease serine protease 2
TNF-α	Tumor necrosis factor-α
TRAF3	TNF Receptor Associated Factor 3
UBE2V1	Ubiquitin-conjugating enzyme E2 variant 1
UNC93B1	Unc-93 homolog B1
UTR	Untranslated region
VEGF	Vascular endothelial growth factor
VWF	von Willebrand factor
WSB1	WD Repeat And SOCS Box Containing 1
XCL1	X-C Motif Chemokine Ligand 1
XCR1	X-C Motif Chemokine Receptor 1
